# Water Dynamics in Whey-Protein-Based Composite Hydrogels by Means of NMR Relaxometry

**DOI:** 10.3390/ijms22189672

**Published:** 2021-09-07

**Authors:** Baris Ozel, Danuta Kruk, Milosz Wojciechowski, Maciej Osuch, Mecit Halil Oztop

**Affiliations:** 1 Department of Food Engineering, Middle East Technical University, Ankara 06800, Turkey; bozel@metu.edu.tr (B.O.); mecit@metu.edu.tr (M.H.O.); 2 Department of Food Engineering, Ahi Evran University, Kirsehir 40100, Turkey; 3 Department of Physics & Biophysics, Faculty of Food Sciences, University of Warmia and Mazury in Olsztyn, Michala Oczapowskiego 4, 10-719 Olsztyn, Poland; maciej.osuch@uwm.edu.pl; 4 Faculty of Mathematics and Computer Science, University of Warmia and Mazury in Olsztyn, Sloneczna 54, 10-710 Olsztyn, Poland; wojciechowski@matman.uwm.edu.pl

**Keywords:** nuclear magnetic resonance, relaxation, hydrogels, whey protein, diffusion, dynamics

## Abstract

Whey-protein-isolate-based composite hydrogels with encapsulated black carrot (*Daucus carota*) extract were prepared by heat-induced gelation. The hydrogels were blended with gum tragacanth, pectin and xanthan gum polysaccharides for modulating their properties. ^1^H spin-lattice relaxation experiments were performed in a broad frequency range, from 4 kHz to 30 MHz, to obtain insight into the influence of the different polysaccharides and of the presence of black carrot on dynamical properties of water molecules in the hydrogel network. The ^1^H spin-lattice relaxation data were decomposed into relaxation contributions associated with confined and free water fractions. The population of the confined water fraction and the value of the translation diffusion coefficient of water molecules in the vicinity of the macromolecular network were quantitatively determined on the basis of the relaxation data. Moreover, it was demonstrated that the translation diffusion is highly anisotropic (two-dimensional, 2D).

## 1. Introduction

Hydrogels are polymeric networks capable of absorbing a high amount of water [1]. The swelling properties of hydrogels are related to chemical or physical crosslinking of water-soluble polymers [2,3,4]. Physical cross-linking occurs as a result of hydrogen bonds and hydrophobic forces that can be affected by factors such as temperature or pH—consequently, physical cross-linking is referred to as reversible [2,4]. In case of covalent bonding, the cross-linking becomes stable and the materials are referred to as chemical hydrogels [1,4,5].

Although hydrogels can include both synthetic and natural biodegradable polymers [1], in food industry there is a growing interest in the production and application of natural biodegradable hydrogels rather than synthetic ones [6]. Polysaccharides, e.g., alginate [7], pectin [8] and chitosan [9], and proteins, e.g., whey [10], soy [11] and pea proteins [12], have been widely used in food-grade hydrogel production. Moreover, two or more different polymers can be blended to obtain hydrogels with distinct characteristics [13].

Blended protein-based composite hydrogels are used for active agent encapsulation and controlled release purposes [13]. Protein-based hydrogels are typically produced by heating protein solutions above the denaturation temperature. The heating facilitates changes in the protein structure (unfolding followed by aggregation) leading to cross-linking. The hydrogels protect encapsulated bioactive agents, ensuring their delivery with minimum damage to a specific location with a desired rate [14]. Encapsulation of nutraceuticals including polyphenol-containing materials in protein-based hydrogels has recently gained interest [15]. An example of materials rich in plant polyphenols is black carrot concentrate (BC); that is, the juice of black carrot plant (*Daucus carota*). BC has high phenolic content and antioxidant capacity [16]. However, degradation of these compounds during controlled release is inevitable, since they are susceptible to changes in environmental conditions [17,18]. In order to prevent the loss of bioactivity and bioavailability of such compounds during processing, digestion or any other food application, BC can be encapsulated in hydrogels [10].

The mechanism and time-scale of the dynamics of water present in hydrogels, being dependent on interactions of water molecules with the surrounding network, affect the physicochemical properties of the hydrogels [19]. These dynamical properties of water can be probed by NMR relaxometry that provides information not available by means of conventional methods such as mass spectroscopy [20], Fourier transform infrared spectroscopy [21] or water activity measurements [22].

The amount and dynamics of water in a food product affect the overall quality and acceptability of that product [23]. NMR relaxometry has been applied to describe water dynamics in complex food systems [24], biopolymer suspensions and hydrogels [25]. Water is expected to form pools of different dynamical properties within the polymer matrices constituting the hydrogel network [26]. A fraction of water molecules interacts with the polymer network and, consequently, the motion of the molecules is affected by these interactions. This effect is reflected by changes in the ^1^H spin-lattice relaxation compared to bulk water [27].

Fast Field Cycling (FFC)-NMR relaxometry enables performing relaxation experiments over a wide range of resonance frequencies [28]. Relaxation processes monitored at low frequencies provide information about the slow molecule dynamics that cannot be probed by classical NMR relaxation measurements performed at a single, high frequency [29]. FFC-NMR relaxometry is a more recent technique with respect to classical NMR experiments. Therefore, there is a limited number of studies in the food science area which facilitated the FFC-NMR technique. Rachocki et al. (2012) applied this method to dry cress seeds [30]. Spin-lattice relaxation measurements by means of FFC relaxometry were also conducted for rape oil [31]. Steele et al. (2016) monitored the aging of banana and spoilage of milk during storage by FFC-NMR relaxometry [32]. Phenolic analysis of the fresh and withered blueberries [33], characterisation of dry cured ham [34] and differentiation of pistachio oils [35] are also among the rare studies that used FFC techniques for food science applications. One should also mention the recent works [36,37,38] devoted to cheese, eggs and gelatin products, respectively, as they include a thorough, quantitative analysis of NMR relaxometry data, showing the potential of this method for food science.

The broad frequency range (4 kHz–40 MHz) of the FFC-NMR method allows a thorough analysis of the relaxation features, making it possible to link macroscopic behavior of the analysed substances with their dynamical properties at the molecular level [39,40,41]. A prominent area of applications of FFC-NMR relaxometry is colloidal dispersions and hydrogels [42]. The type of the used polymers and their concentration, the presence of ions or additional stabilisers and encapsulation of active agents are among the factors that influence water dynamics in hydrogel systems [43].

The main objective of this study is to observe the effects of different hydrogel formulations on the dynamics of water molecules enclosed into those systems. For this purpose, whey-protein-isolate (WPI)-based hydrogels were produced by blending polysaccharides including gum tragacanth (GT), pectin (PC) and xanthan gum (XG). BC was encapsulated in these protein–polysaccharide hydrogels. FFC-NMR relaxometry was applied to enquire into the influence of the hydrogel composition and the presence of BC on water mobility.

## 2. Results

### 2.1. Theoretical Model of ^1^H Spin-Lattice Relaxation

In macromolecular systems (food products being an example of them) one can distinguish two water molecule fractions, often defined as the free-water fraction and confined-water fraction. The two pools of water molecules differ in terms of their dynamical properties: the dynamics of water molecules representing the free-water fraction is similar to the dynamics of water in bulk, while the motion of the molecules of the confined-water pool is slower and often restricted (anisotropic) by the macromolecular network forming the confinement. Consequently, the overall ^1^H spin-lattice relaxation rate, $R_{1,H}\left(\omega_{H}\right)$ ($\omega_{H}$ displays ^1^H resonance frequency), is given as a sum of two contributions [44]:

(1) $$\;R_{1,H}\left(\omega_{H}\right)=R_{1,H}^{conf}\left(\omega_{H}\right)+R_{1,H}^{free}\left(\omega_{H}\right)\;$$

The relaxation contribution associated with the free-water fraction, $R_{1,H}^{free}\left(\omega_{H}\right)$, could be represented by a frequency-independent term, $R_{1,H}^{free}\left(\omega_{H}\right)=A$, as a result of the fast motion of water molecules belonging to that fraction. ^1^H spin-lattice relaxation is predominantly caused by magnetic dipole–dipole interactions that can be of inter-molecular and intra-molecular origin. Thus, the relaxation contribution, $R_{1,H}^{conf}\left(\omega_{H}\right)$, includes relaxation terms $R_{1,H}^{conf,\;inter}\left(\omega_{H}\right)$ and $R_{1,H}^{conf,\;intra}\left(\omega_{H}\right)$:

(2) $$R_{1,H}^{conf}\left(\omega_{H}\right)=R_{1,H}^{conf,\;inter}\left(\omega_{H}\right)+R_{1,H}^{conf,\;intra}\left(\omega_{H}\right)\;$$

The inter-molecular dipole–dipole interactions are modulated in time due to translation diffusion of the water molecules. The form of the relaxation contribution depends on the dimensionality of the translation motion. In bulk water, molecules can freely (isotropically) move in all directions, so the diffusion process is three-dimensional (3D). An indication of 3D character of the translation dynamics is a linear dependence of the relaxation rate, $R_{1,H}\left(\omega_{H}\right)$, on the square root of the resonance frequency, $\sqrt{\omega_{H}}$, observed at low frequencies [45]. Isotropic (3D) translation diffusion can also be observed in confinement, but then the linear dependence can be, to some extent, masked by other relaxation contributions ($R_{1,H}^{conf,\;intra}\left(\omega_{H}\right)$ in this case). In confinement, one can, however, also expect that the translation motion of molecules belonging to the confined-water fraction is of rather two-dimensional (2D) character—the molecules move on the surface (in the vicinity) of the macromolecular network. An indication of the 2D mechanism of the translation diffusion is a linear dependence of $R_{1,H}\left(\omega_{H}\right)$ on $ln\left(\omega_{H}\right)$ in the low frequency range [46]. Again, this effect can be masked by other contributions, so one might not be able to see it directly, but for the relaxation data obtained for the hydrogel system one can identify a frequency range in which the relaxation rate, $R_{1,H}\left(\omega_{H}\right)$, depends linearly on $ln\left(\omega_{H}\right)$. For 2D translation diffusion, the relaxation contribution $R_{1,H}^{conf,\;inter}\left(\omega_{H}\right)$ can be expressed as:

(3) $$R_{1,H}^{conf,\;inter}\left(\omega_{H}\right)=C_{DD}^{inter}\tau_{trans}^{conf}\left[ln\left(1+\frac{1}{{\left(\omega_{H}\tau_{trans}^{conf}\right)}^{2}}\right)+4ln\left(1+\frac{1}{{\left(2\omega_{H}\tau_{trans}^{conf}\right)}^{2}}\right)\right]\;$$

where $C_{DD}^{inter}$ is referred to as an inter-molecular dipolar relaxation constant, while the correlation time $\tau_{trans}^{conf}$ is defined as: $\tau_{trans}^{conf}=\frac{d^{2}}{2D_{trans}^{conf}}$ and $D_{trans}^{conf}$ denotes the translation diffusion coefficient of water molecules constituting the confined-water fraction. Additionally, d is the diameter of the water molecule. In fact, the model includes a parameter referred to as “the distance of closest approach”. This parameter is well-defined for spherical molecules with ^1^H nuclei placed in their center—then the minimum ^1^H-^1^H distance is equal to the molecular diameter. For “real” molecules, this approximation is also realistic. In the low frequency range, in which the condition $\omega_{H}\tau_{trans}^{conf}$ < 1 is fulfilled, Equation (3) can be approximated as:

(4) $$R_{1,H}^{conf,\;inter}\left(\omega_{H}\right)\propto \left[-\tau_{trans}^{conf}ln\left(\omega_{H}\tau_{trans}^{conf}\right)\right]\;$$

Equation (4) explains the statement above about a linear dependence of $R_{1,H}\left(\omega_{H}\right)$ on $ln\left(\omega_{H}\right)$ for 2D translation diffusion. The relaxation constant $C_{DD}^{inter}$ is proportional to the number of water molecules per unit volume in the confined-water fraction. The relaxation contribution $R_{1,H}^{conf,\;intra}\left(\omega_{H}\right)$ is associated with rotational dynamics and it can be described as [47]:

(5) $$R_{1,H}^{conf,intra}\left(\omega_{H}\right)=C_{DD}^{intra}\left[\frac{\tau_{c}}{1+{\left(\omega_{H}\tau_{c}\right)}^{2}}+\frac{4\tau_{c}}{1+{\left(2\omega_{H}\tau_{c}\right)}^{2}}\right]\;$$

The intra-molecular dipolar relaxation constant, $C_{DD}^{intra}$, representing the water molecules bound to macromolecules, is proportional to the product Pq. Here, q and P stand for the coordination number and the mole fraction of water protons in the bound position, respectively [48]. Water molecules form a kind of hydration shell (similarly to, for instance, protein solutions). This effect is likely related to hydrogen bounds involving OH groups of the polysaccharides. The rotational correlation time of a macromolecule having water molecules in bound position, $\tau_{rot}$, determines the correlation time $\tau_{c}$. Then, the exchange lifetime of water molecules in the bound position can be denoted as $\tau_{ex}$: $\tau_{c}^{-1}=\tau_{rot}^{-1}+\tau_{ex}^{-1}$.

### 2.2. Relaxation Data

**Figure 1:**
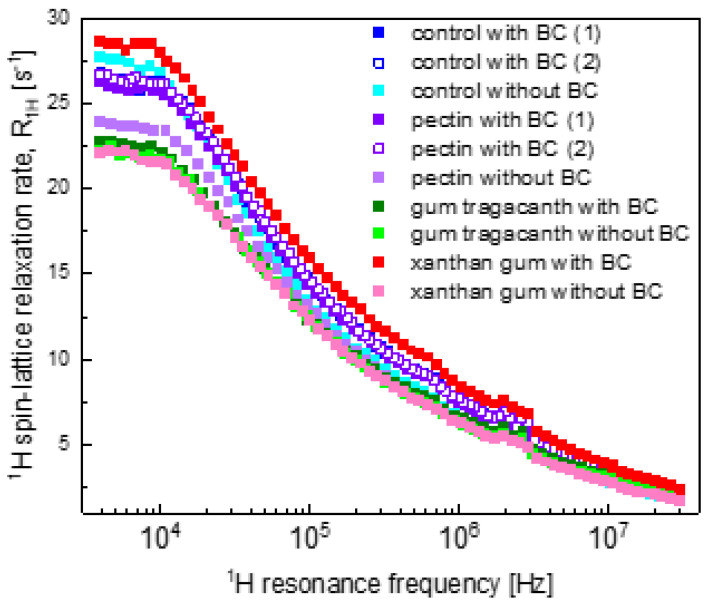
^1^H spin-lattice relaxation data for all hydrogel systems. The largest uncertainty of the values is 5.6%; for the majority of the relaxation rates the uncertainty is of the order of 3%.

The hydrogel formulations are summarised in Table 1. In Figure 1, ^1^H spin-lattice relaxation data for all systems are collected for comparison. The data have been reproduced in terms of the theoretical model of ^1^H spin-lattice relaxation presented in Section 2.1. Below, we compare and discuss the relaxation data step-by-step, beginning with the control hydrogel (C) systems.

In Figure 1, one sees two data sets for the control samples containing BC (referred to as “control with BC(1) and BC(2)”) and the pectin samples containing BC (referred to as “pectin with BC(1) and BC(2)”). The data were collected for two samples of the same formulation to show reproducibility of the results.

**Figure 2:**
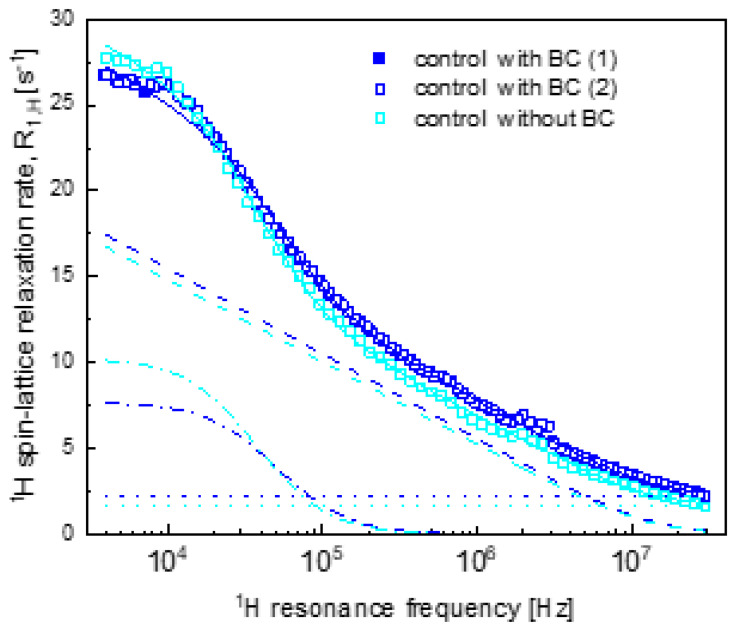
^1^H spin-lattice relaxation rates for control hydrogels (control) with BC and without BC. Solid lines—corresponding fits ($R_{1,H}\left(\omega_{H}\right))$ for the control samples decomposed into $R_{1,H}^{conf,\;inter}\left(\omega_{H}\right)$—dashed lines, $R_{1,H}^{conf,\;intra}\left(\omega_{H}\right)$ —dashed–dotted lines and A —dotted lines.

Figure 2 shows a ^1^H spin-lattice relaxation curve for the control hydrogels without BC and with BC. As expected, the parameters for the control systems do not differ much (Table 2). The translation diffusion coefficients are of the order of 1 × 10^−11^ m^2^/s, which makes the translation diffusion by about 500 times slower compared to water in bulk. The correlation time $\tau_{c}$ is of the order of 2 × 10^−6^ s. The similar values of the dipolar relaxation constant $C_{DD}^{intra}$ indicate that the mole fractions of water molecules bound to the macromolecular network are very similar in both cases, while the slightly different values of $C_{DD}^{inter}$ indicate that the number of water molecules per unit volume in the confined-water fraction is somewhat different.

**Figure 3:**
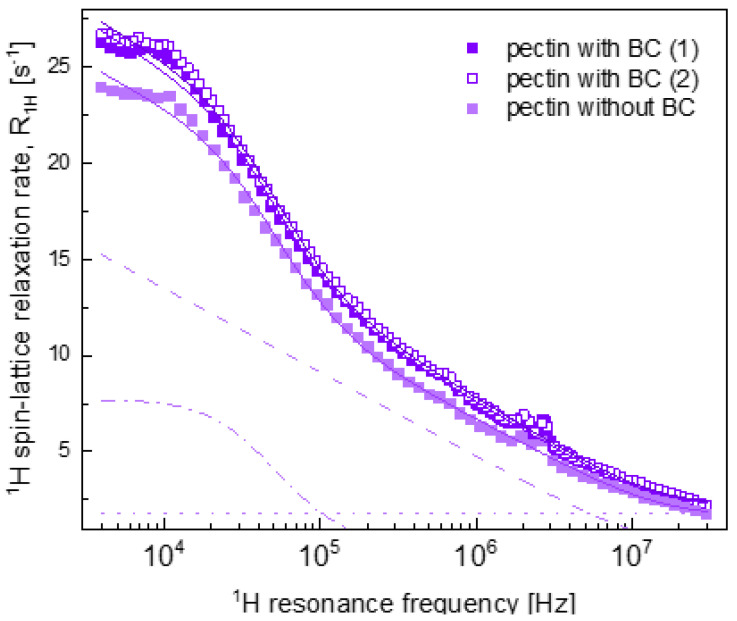
^1^H spin-lattice relaxation data for pectin hydrogels with and without BC. Solid and double solid violet lines—Figure 1. of the data for pectin with BC (1) and (2) data sets, respectively, solid light violet line—fit ($R_{1,H}\left(\omega_{H}\right))$ of the data for pectin without BC decomposed into $R_{1,H}^{conf,\;inter}\left(\omega_{H}\right)$—dashed line, $R_{1,H}^{conf,\;intra}\left(\omega_{H}\right)$—dashed–dotted line and A —dotted line.

The results of the analysis of the relaxation data for PC hydrogels with and without BC (the overall fit decomposed into the individual contributions) are shown in Figure 3. The relaxation data for PC with BC hydrogels are in good agreement with each other (the sample preparation and the relaxation measurements are reproducible) and they are very close to the relaxation data for the control system with BC. Consequently, the obtained parameters (included in Table 2) are very similar to those for the control system—the corresponding fits are shown in Figure 3. At the same time, the relaxation data for PC without BC hydrogel considerably differ from the case when BC is present and, consequently, from the relaxation data for the control hydrogel system without BC. The results of the analysis of the relaxation data for PC without BC hydrogel (the overall fit decomposed into the individual contributions) are also shown in Figure 3.

**Figure 4:**
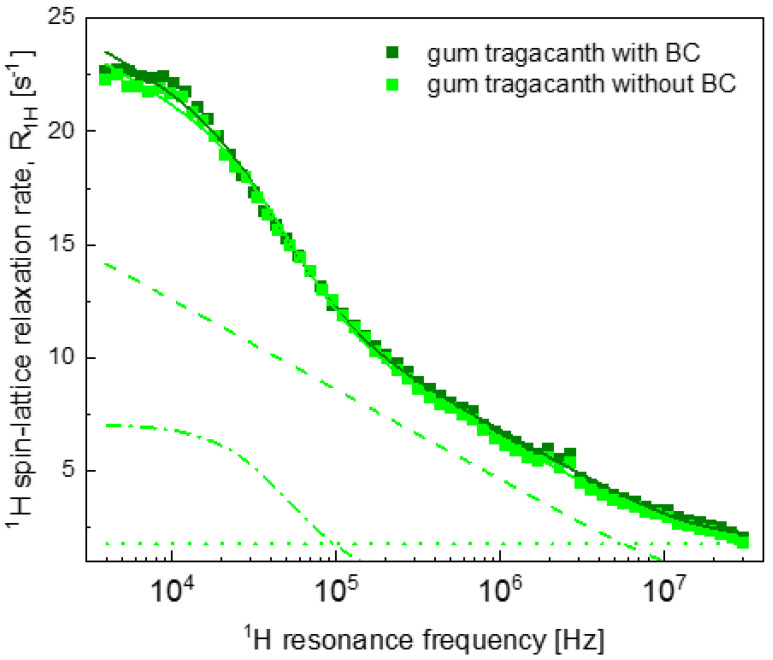
^1^H spin-lattice relaxation data for gum tragacanth hydrogels with and without BC. Solid lines—fits ($R_{1,H}\left(\omega_{H}\right))\;$of Table 1. dashed line, $R_{1,H}^{conf,\;intra}\left(\omega_{H}\right)$—dashed–dotted line and A —dotted line.

Figure 4 shows the analysis for the GT hydrogels with and without BC—the relaxation data for these systems are very similar.

**Figure 5:**
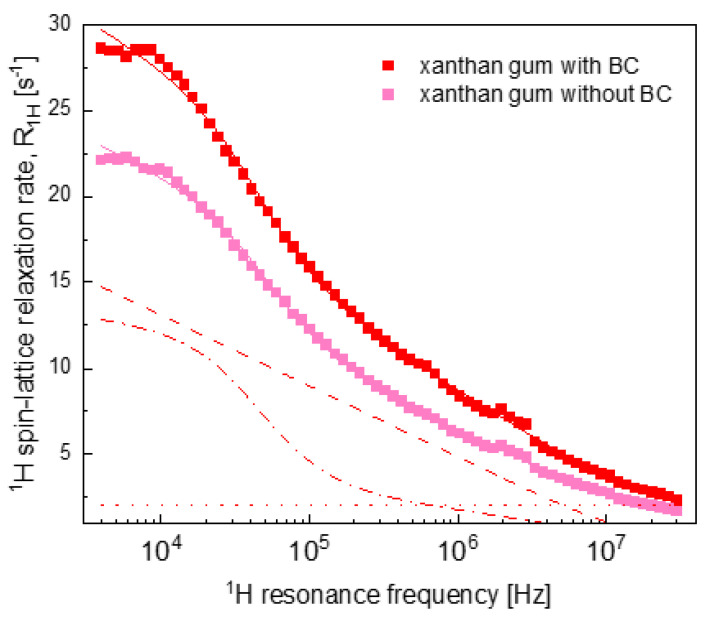
^1^H spin-lattice relaxation data for xanthan gum hydrogels with and without BC. Solid lines—fits ($R_{1,H}\left(\omega_{H}\right))\;$of the data decomposed (for xanthan system with BC) into: $R_{1,H}^{conf,\;inter}\left(\omega_{H}\right)$—dashed line, $R_{1,H}^{conf,\;intra}\left(\omega_{H}\right)$—dashed–dotted line and A —dotted line.

Figure 5 displays the overall relaxation data for the XG hydrogel systems. The XG hydrogel without BC exhibits similar parameters to the GT systems (as expected from Figure 1). At the same time, the relaxation rates for the XG with BC hydrogels are considerably different from the data for the rest of the samples.

The parameters obtained from the analysis of the data shown in Figure 4 and Figure 5 are also included in Table 2.

## 3. Discussion

Comparing the obtained parameters, one can observe that the difference in the relaxation properties between the PC without BC hydrogel system and the other systems (Figure 1) was caused by a lower $C_{DD}^{inter}$ value for the PC without BC system. This result indicates a smaller number of water molecules per unit volume in the confined-water fraction in the absence of BC. BC is a rich source of anthocyanins [49] that can form hydrogen bonds with the polymers constituting the hydrogel structure [50]. It seems that in the presence of BC, a larger fraction of the confined water molecules gained the freedom of translation motion. One should note that with the exception of the control system (Figure 2), the other systems (PC, GT and XG) without BC are characterised by a lower value of $C_{DD}^{inter}$ than for their counterparts with BC; for the control hydrogel system, the relationship is the opposite. Since the control hydrogels are solely composed of WPI, the lack of an additional polysaccharide that would contribute to the polymer–water interactions [51] likely decreases the effect of BC on the formation of the gel network. 

The obtained parameters for GT hydrogel systems indicate that in the case of GT hydrogel, relatively small relaxation rates in the low frequency range (Figure 1) rather originate from faster translation diffusion of water molecules in the confined-water fraction than from lower values of the number of water molecules per unit volume in this fraction (although, as already pointed out, the value is lower in the absence of BC). Additionally, the number of water molecules in the bound position (reflected by the $C_{DD}^{intra}$ value) is somewhat smaller. GT is a physical mixture of bassorin and tragacanthin, water-swellable and water-soluble fractions, respectively [52]. The presence of a tragacanthin fraction imparts upon GT a liquid character. Tragacanthin includes sugar moieties, increasing the hydrophilic character of the polysaccharide. Consequently, one can suppose that the dynamical features of water, affected by interactions of water molecules with tragacanthin, make the effects of adding BC considerably less significant. It is likely that the faster translation movement is related to the texture of the GT hydrogels. Ozel et al. (2018) demonstrated that GT hydrogels had lower hardness values with respect to the control hydrogel systems [53]. 

The distinct relaxation features of XG with BC hydrogels (Figure 5) are reflected by the set of parameters for this system presented in Table 2. The dipolar relaxation constant $C_{DD}^{inter}$ for the XG with BC system was significantly larger than for the other systems, indicating a higher population of the sub-fraction of the confined-water pool that performs translation diffusion—this effect manifested itself by the high ^1^H spin-lattice relaxation rates for the XG system with BC at low frequencies. This different behaviour of XG with BC hydrogels may originate from the highly branched complex structure of XG molecules [54]. The molecular weight (M_w_) of XG (~2000 kDa) [55] is also higher than the M_w_ of the other polysaccharides, PC (~100 kDa) [56] and GT (~850 kDa) [57], used in the study. The branched side chains of XG molecules may have created hydrogen bonds with the anthocyanins of the BC, which resulted in better incorporation of BC into the gel network. As a result, the availability of translation diffusion paths for the water molecules may increase. It is worth mentioning that even at low concentrations (<1%), XG substantially increases the solution viscosity [58]. Moreover, BC was also reported to increase the viscosity of protein solutions. Ozel et al. (2018) stated that for the same set of hydrogels, only XG with BC hydrogel solutions exerted pseudoplastic behaviour whereas all other hydrogel solutions (C, PC and GT, all with BC) had Newtonian flow behaviour [53]. Such a behaviour was not observed for the XG without BC hydrogels and the viscosity increasing effect of BC was not present for these samples. This observation is in agreement with the results of the other polysaccharide blended hydrogels with no BC.

Besides the differences in the ^1^H spin-lattice relaxation rates of the samples, all the relaxation data show relaxation maxima (weakly pronounced) in the frequency range of 2–3 MHz (Figure 1). These relaxation maxima originate from the Quadrupole Relaxation Enhancement (QRE) effect, and the maxima are called quadrupole peaks [37,59]. They stem from ^1^H-^14^N dipole–dipole interactions and can be observed for systems undergoing relatively slow dynamics [37]. In fact, for ^14^N one can observe three quadrupole peaks, the first one at a frequency being a difference between the frequencies at which the other two are present [60]. Therefore, we suppose that the QRE effect somewhat affects the relaxation data at low frequencies. As it is difficult to exactly specify the ^1^H–^14^N relaxation contribution (in principle one could model this effect [37,61], but this would require involving several parameters that can hardly be verified), the QRE effect is likely the reason of some discrepancies between the fits and the experimental data at low frequencies.

Finally, one should point out the two-dimensional character of the translation motion of water in the hydrogel network, although the network itself is three-dimensional. This finding gives an important insight into the scenario of the translation movement of water molecules confined in food hydrogels.

## 4. Materials and Methods

### 4.1. Materials

Whey protein isolate (WPI) with 88.5% (*w*/*w*) protein content (Kavi Food Ltd. Co., Istanbul, Turkey), high methoxy citrus pectin (PC) (FMC SRL, San Colombano al Lambro, Italy) with an esterification degree around 64–68%, gum tragacanth (GT) from *Astragalus gummifier* Labillardiere having 40% bassorin and 60% tragacanthin composition (*w*/*w*) (Thew Arnott & Co. Ltd., Deeside, UK) and xanthan gum (XG) from *Xanthomonas campestris* (Smart Kimya Tic. ve Danismanlik Ltd. Sti., Izmir, Turkey) were the agents used for gelation and hydrogel production. Black carrot extract (BC) (Targid Agriculture Co. Inc., Icel, Turkey) was the bioactive agent encapsulated in the hydrogels. Sodium azide (Merck KgaA, Darmstadt, Germany) was included in the all-hydrogel formulations to prevent microbial activity.

### 4.2. Sample Preparation

Initially, 15% (*w*/*w*) WPI solutions were prepared and PC, GT or XG polysaccharides were added to these protein solutions so that the final polysaccharide concentration reached 0.5% (*w*/*w*). Polysaccharide-containing WPI hydrogels were denoted as PC, GT and XG hydrogels depending on the polysaccharide used in the formulation. Sole WPI-containing samples (C hydrogels) were also prepared with no additional polysaccharide. As a bioactive agent, 4% (*w*/*w*) BC was added to hydrogel-forming solutions. The same set of hydrogels was also prepared in the absence of BC. Water was added to these BC-free samples to compensate for the amount of BC in these formulations. 

Polysaccharide solutions were mixed with BC and stirred at 15,000 rpm for 2 min by an Ultra Turrax T-18 (IKA Corp., Staufen, Germany). WPI–sodium azide solutions were also stirred in the same way, separately. Then, protein and polysaccharide–BC solutions were mixed in a flask and stirred overnight at room temperature for the complete hydration of the polymers. BC-containing solutions had lower pH values (5.7–5.9) than the ones without BC (6.8–7.0) at room temperature. The next day, hydrogel solutions were placed into cylindrical gelling tubes (1.5 cm outer diameter, 1.3 cm inner diameter, 5 cm length) and put into a water bath (Wisd, Wertheim, Germany) operating at 90 °C for 30 min to achieve gelation. After gelling in the water bath, cylindrical tubes were immersed in ice for 15 min and then hydrogels were extracted from the tubes. 

### 4.3. Fast Field Cycling (FFC) NMR Relaxometry Measurements

Small cylindrical pieces from the hydrogels (2 cm length × 0.65 cm diameter) were cut and put into tubes that were used for the NMR relaxometry measurements. ^1^H spin-lattice relaxation rates of the hydrogel samples were measured with an FFC Spinmaster 2000 Relaxometer (Stelar, Mede, Italy) and the range of ^1^H resonance frequencies covered in the experiment was from 4 kHz to 30 MHz. During the measurements, a Eurotherm controller with an additional thermocouple temperature sensor inserted into the probe-head was used to control the temperature with the accuracy of 0.5 °C. The relaxation process turned out to be single-exponential. Examples of ^1^H magnetisation curves (^1^H magnetisation value versus time) accompanied with single-exponential fits are shown in the Supplementary Materials.

## 5. Conclusions

^1^H spin-lattice relaxation data for a series of composite WPI hydrogels collected in a wide frequency range (4 kHz to 30 MHz) were quantitatively analysed. On this basis, two-dimesional translation motion of water molecules in the hydrogel network was revealed, although the network itself is three-dimensional, and parameters characterising translation diffusion of water molecules ($\tau_{trans}^{conf}$ and $D_{trans}^{conf}$) were obtained. It turned out that the translation diffusion of water molecules belonging to the confined-water fraction was slowed down in all hydrogels by about 500 times with respect to that of bulk water. The translation diffusion coefficients for all systems turned out to be in the order of 1 × 10^−11^ m^2^/s, while the correlation time $\tau_{c}$ was in the order of 2 × 10^−6^ s. This long correlation time indicates that the water molecules undergo a tumbling (rotation) together with the macromolecules to which they are bound. The similar values of the intra-molecular dipolar relaxation constant $C_{DD}^{intra}$ imply that, whether BC is present or not, the product *Pq* is similar. One should here clearly distinguish between the $C_{DD}^{intra}$ and $C_{DD}^{inter}$ quantities—the first one carries information about the sub-fraction of water molecules within the confined-water fraction that are bound to the macromolecular structure, while the second one gives inside information into the population of the sub-fraction of the confined-water pool that performs translation diffusion. The similar parameters characterising water dynamics point towards generic features of different food hydrogels in terms of the water mobility. The motion of water molecules in the macromolecular network is an important factor determining the macroscopic properties of hydrogels, such as their viscosity, elasticity, hardness or ability to hold and release their cargo.

In this work, we provide insight into the dynamical properties of water dynamics in food hydrogels, exploiting the unique potential of NMR relaxometry. Thinking about exploiting this kind of information for tailoring properties of food hydrogels, the closest systems of interest are confectionary gels, candies and some hydrocolloid solutions.

## Figures and Tables

**Table 1:** Formulations of hydrogels in the presence and absence of BC. (“C”, “PC”, “GT” and “XG” denote control, pectin, gum tragacanth and xanthan gum, respectively).

Hydrogels	Water (%) *	BC (%)	WPI (%)	PC (%)	GT (%)	XG (%)
C with BC	81	4	15	-	-	-
PC with BC	80.5	4	15	0.5	-	-
GT with BC	80.5	4	15	-	0.5	-
XG with BC	80.5	4	15	-	-	0.5
C without BC	85	-	15	-	-	-
PC without BC	84.5	-	15	0.5	-	-
GT without BC	84.5	-	15	-	0.5	-
XG without BC	84.5	-	15	-	-	0.5

* All percentages are (*w*/*w*) basis.

**Table 2:** Parameters obtained from the analysis of ^1^H spin-lattice relaxation data of the hydrogel samples. For the control sample with BC, only one set of parameters is given as the data for BC(1) and BC(2) overlap within the experimental error.

Sample	$C_{DD}^{inter}$ (10^7^ Hz^2^)	$\tau_{trans}^{conf}$ (10^−9^ s)	$D_{trans}^{conf}$ (10^−11^ m^2^/s) *	$C_{DD}^{intra}$ (10^5^ Hz^2^)	$\tau_{c}$ (10^−6^ s)	A (s^−1^)	Relative Error (%)
C with BC	2.87	7.24	1.08	8.57	2.39	1.6	3.1
C without BC	3.14	6.85	1.14	8.72	1.76	2.2	4.3
PC with BC (1)	3.12	6.65	1.18	9.14	1.72	2.1	3.7
PC with BC (2)	3.23	6.56	1.20	9.34	1.69	2.2	3.3
PC without BC	2.58	7.33	1.07	9.12	1.58	1.8	3.9
GT with BC	2.94	5.91	1.33	7.35	1.96	2.0	3.6
GT without BC	2.77	6.20	1.26	8.32	1.69	1.8	3.7
XG with BC	4.33	5.41	1.45	8.28	1.95	2.1	2.9
XG without BC	2.97	6.02	1.33	7.75	1.70	1.6	5.1

* The translation diffusion coefficients were calculated from the relationship $\tau_{trans}^{conf}=\frac{d^{2}}{2D_{trans}^{conf}}$, *d* = 2.8Å (d denotes the diameter of water molecule).

## Supplementary Materials

The following are available online at https://www.mdpi.com/article/10.3390/ijms22189672/s1.
