# Supplementary material for: Water Dynamics in Whey-Protein-Based Composite Hydrogels by Means of NMR Relaxometry
Source: Int J Mol Sci. 2021 Sep 7;22(18):9672. doi: 10.3390/ijms22189672 (PMC8469572; doi:10.3390/ijms22189672)
Supplement: Supplementary file 1 [file ijms-22-09672-s001.zip › ijms-1334025-supplementary.pdf]

Supplementary Material

^{1}H magnetization curves (magnetization versus time) for hydrogels.

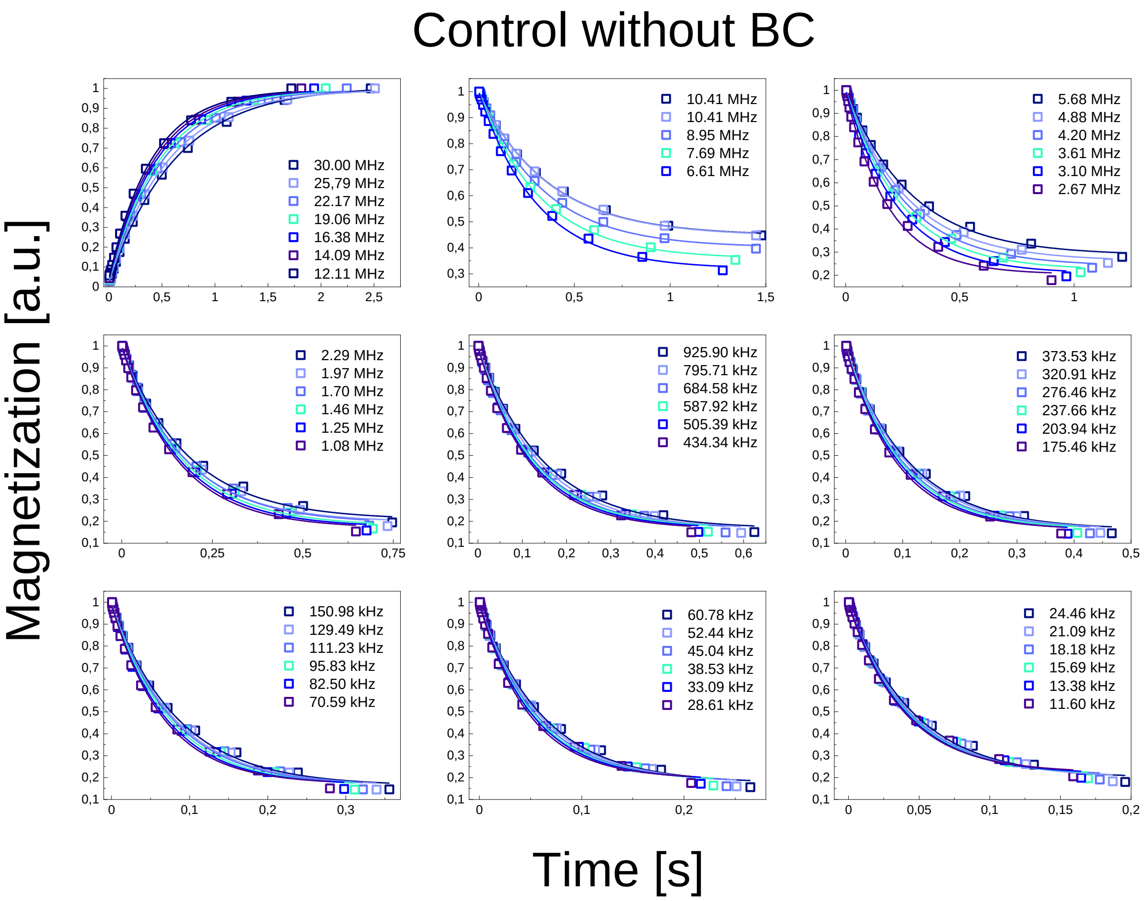

Figure S1. ^{1}H magnetization versus time for the control system without black carrot concentrate.

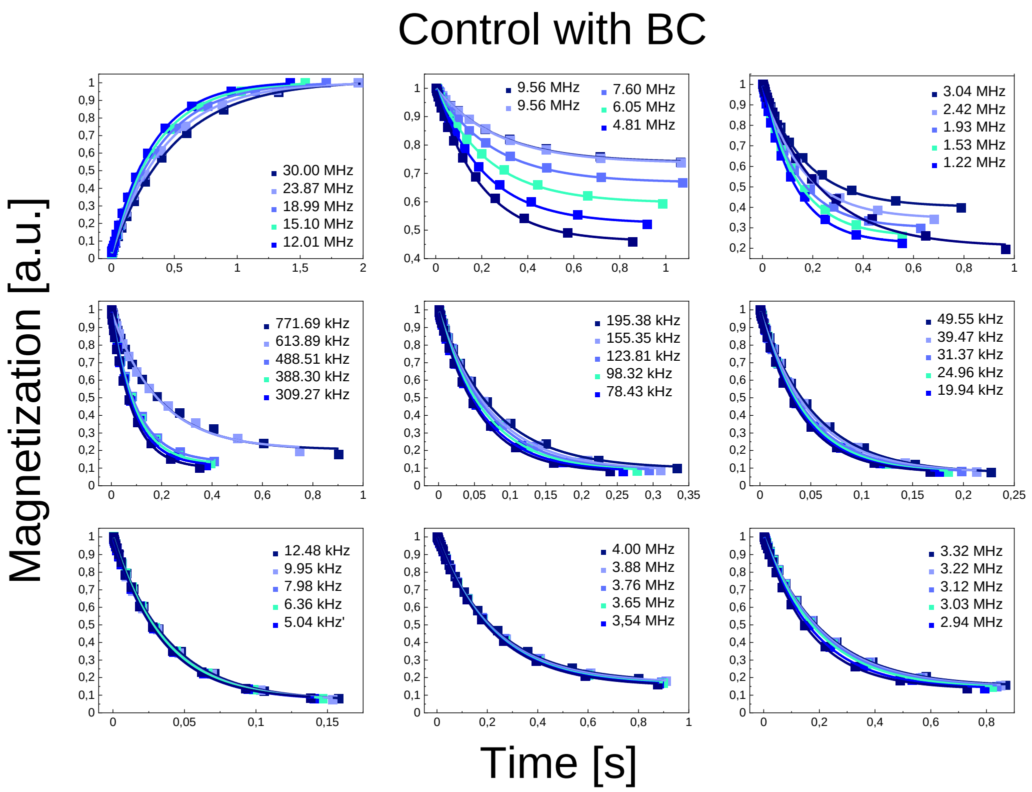

Figure S2. [1]H magnetization versus time for the control system with black carrot concentrate.

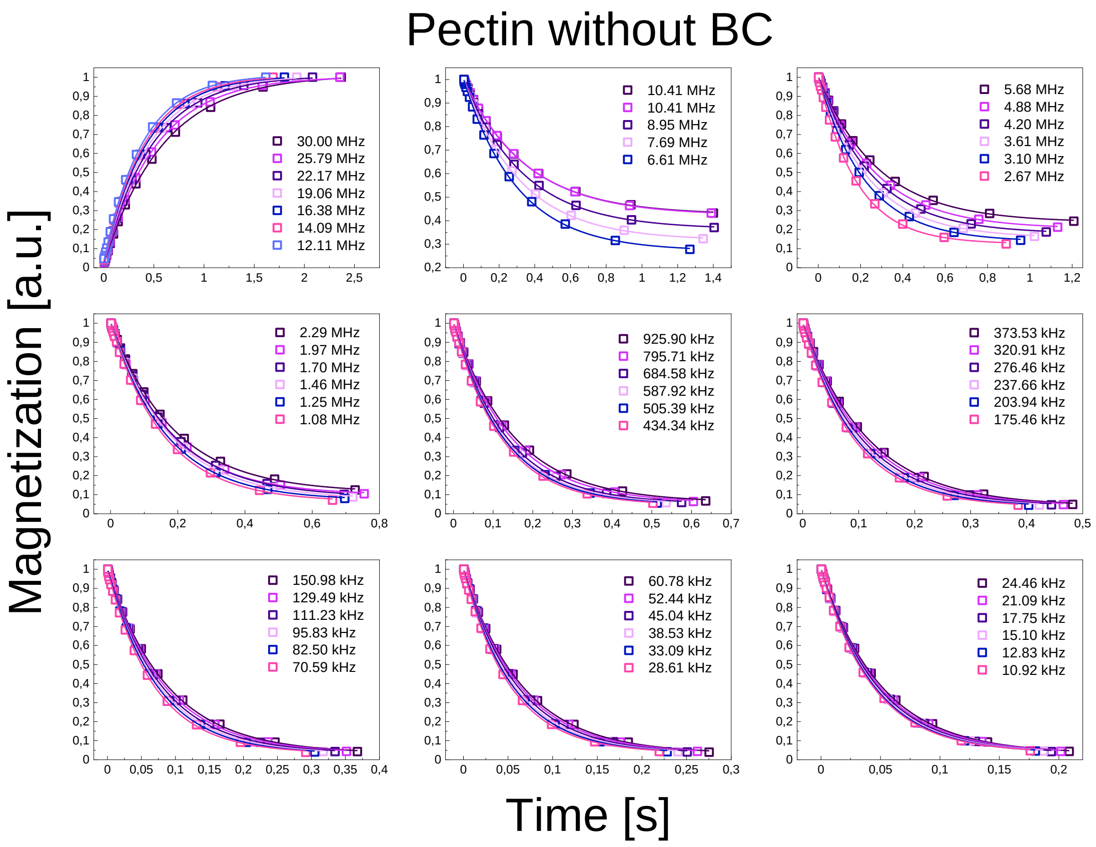

Figure S3. ^{1}H magnetization versus time for the pectin without black carrot concentrate system.

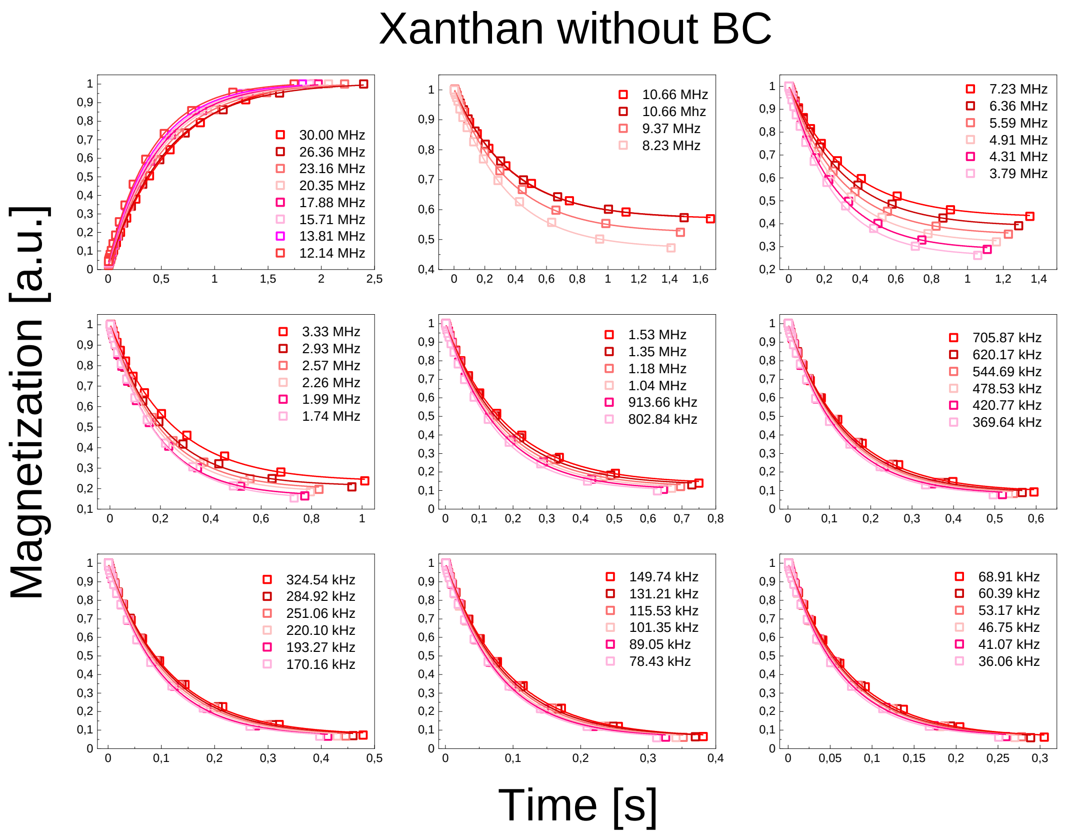

Figure S4. ^{1}H magnetization versus time for the xanthan without black carrot concentrate system.

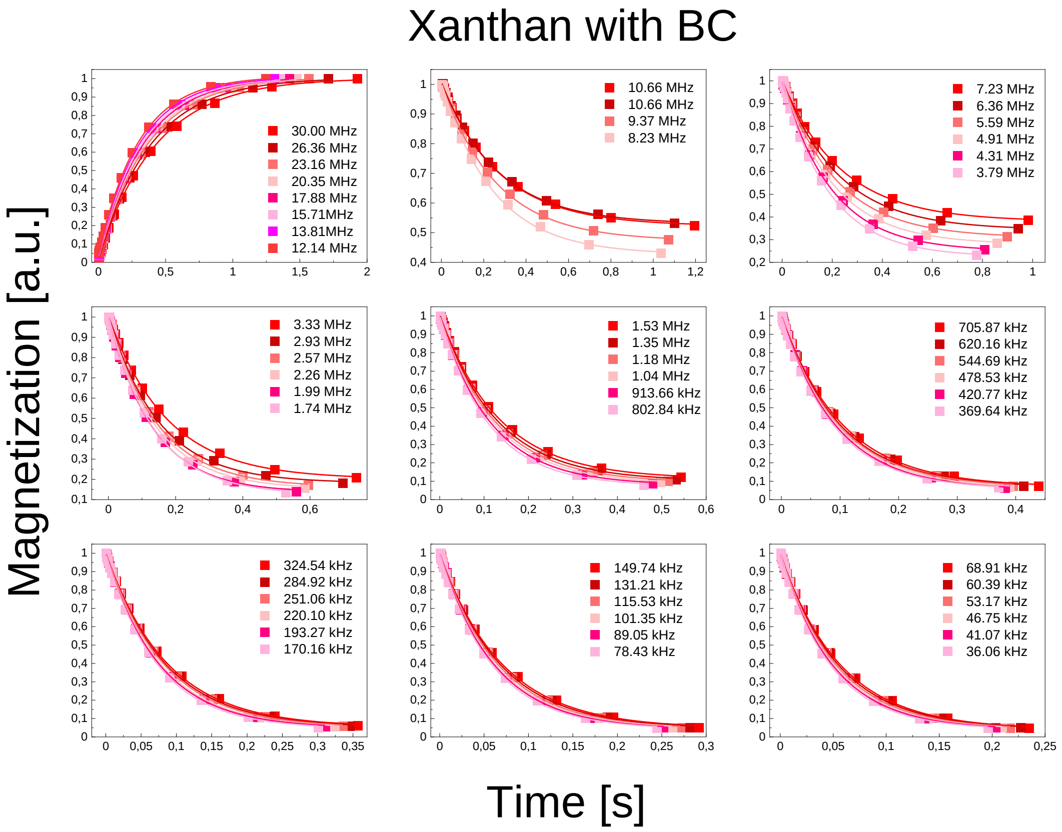

Figure S5. [1]H magnetization versus time for the xanthan with black carrot concentrate system.

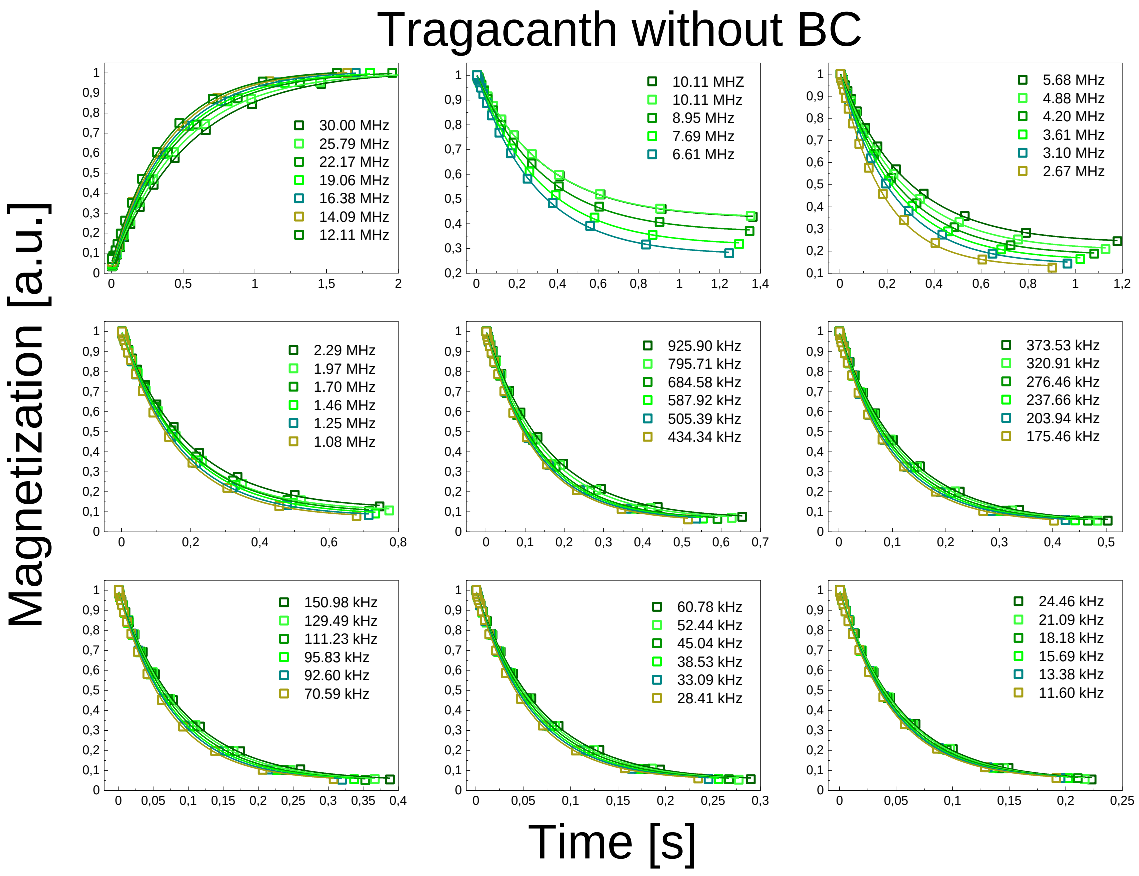

Figure S6. ^{1}H magnetization versus time for the tragacanth without black carrot concentrate system.

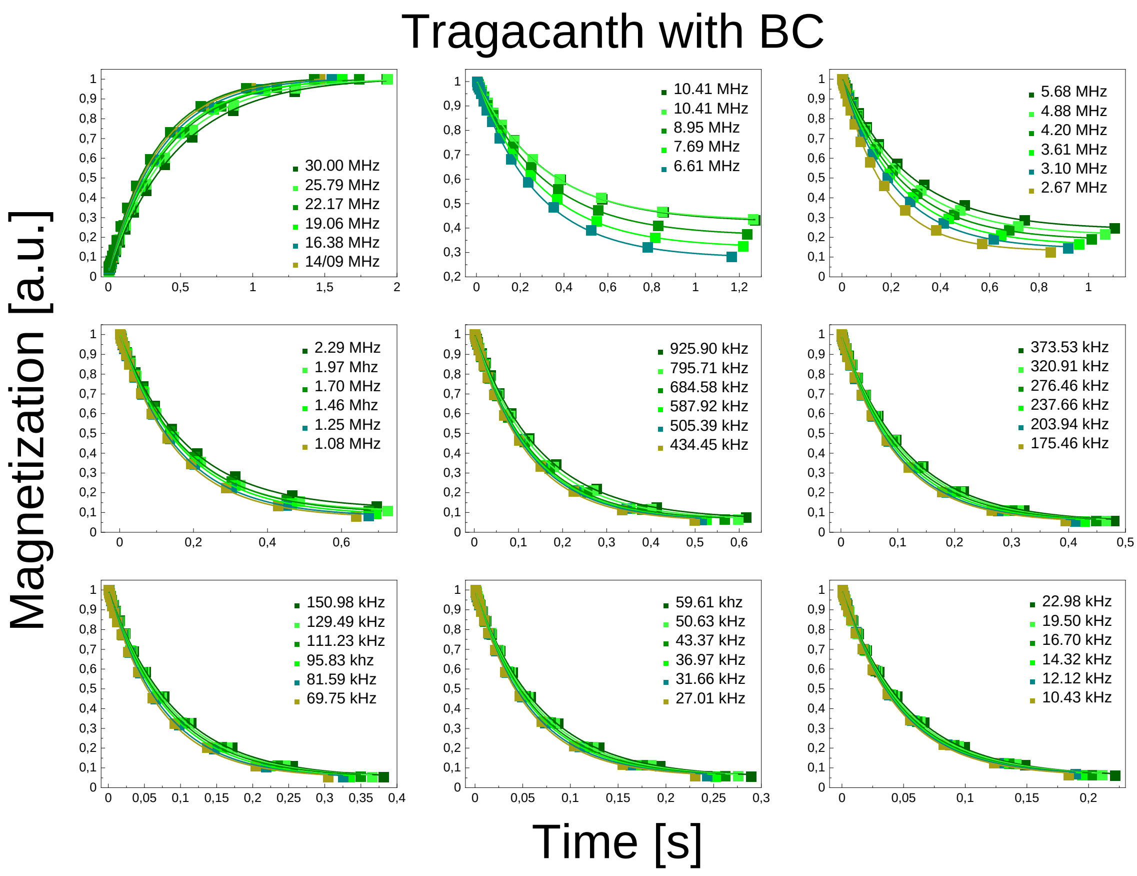

Figure S7. [1]H magnetization versus time for the tragacanth with black carrot concentrate system.
